# Malaria Policy Advisory Committee to the WHO: conclusions and recommendations of sixth biannual meeting (September 2014)

**DOI:** 10.1186/s12936-015-0623-5

**Published:** 2015-03-10

**Authors:** WHO Malaria Policy Advisory Committee and Secretariat

**Affiliations:** Global Malaria Programme, World Health Organization, 20 Avenue Appia, 1211 Geneva 27, Switzerland

**Keywords:** WHO, Malaria, Policy making, Mosquito control, Drug resistance, Surveillance, Elimination, Plasmodium falciparum, Plasmodium vivax

## Abstract

The Malaria Policy Advisory Committee to the World Health Organization held its sixth meeting in Geneva, Switzerland from 10 to 12 September 2014. This article provides a summary of the discussions, conclusions and recommendations from that meeting.

Meeting sessions covered the following: an update on drug resistance and containment including an assessment on the feasibility of elimination of *Plasmodium falciparum* malaria in the Greater Mekong Subregion; guidance on the control of residual malaria transmission by behaviourally resistant vectors; progress on the implementation of the Global Plan for Insecticide Resistance Management; updates on the Global Technical Strategy, Global Malaria Action Plan and the *Plasmodium vivax* technical brief; gaps in current World Health Organization Global Malaria Programme guidance for acceleration to elimination; surveillance, monitoring and evaluation; the updated World Health Organization Guidelines for the Prevention and Treatment of Malaria; Round 5 product testing for rapid diagnostic tests; and Intermittent Preventive Treatment for infants.

Policy statements, position statements, and guidelines that arise from the Malaria Policy Advisory Committee meeting conclusions and recommendations will be formally issued and disseminated to World Health Organization Member States by the World Health Organization Global Malaria Programme.

## Background

The Malaria Policy Advisory Committee (MPAC) to the WHO held its sixth meeting from 10 to 12 September 2014 in Geneva, Switzerland, following its meetings in February and September 2012, March and September 2013, and March 2014 [1-5]. This article provides a summary of the discussions, conclusions and recommendations from that meeting^a^ as part of the *Malaria Journal* thematic series “WHO global malaria recommendations” [6].

The following sections of this article provide details and references for the background documents presented at the open sessions of the committee on: an update on drug resistance and containment, including an assessment on the feasibility of *Plasmodium falciparum* malaria elimination in the Greater Mekong Subregion; guidance on the control of residual malaria transmission by mosquitoes whose behaviour, such as outside biting, makes them poorly susceptible to control through Indoor Residual Spraying (IRS) or long-lasting insecticidal nets (LLINs); progress on the implementation of the Global Plan for Insecticide Resistance Management; updates on the Global Technical Strategy, Global Malaria Action Plan and the *Plasmodium vivax* technical brief; gaps in current World Health Organization Global Malaria Programme guidance for acceleration to elimination; surveillance, monitoring and evaluation; the updated World Health Organization Guidelines for the Prevention and Treatment of Malaria; Round 5 product testing for rapid diagnostic tests; and Intermittent Preventive Treatment for infants.

The MPAC discussion and recommendations related to these topics, which took place partially in closed session, are also included. MPAC decisions are reached by consensus [7]. The next meeting of the MPAC will be 4 to 6 March 2015 [8].

### Report from the WHO global malaria programme

The acting Director of the WHO Global Malaria Programme (WHO-GMP) provided updates on behalf of the WHO Americas, African, Eastern Mediterranean, European, South-East Asian, and Western Pacific Regional Offices [9]. All regions reported significant progress, but mentioned important challenges, such as insufficient human resources which can lead to gaps in technical capacity. This lack of human resource across many technical areas in malaria programmes is an area of concern to MPAC members, and has been specifically noted in the field of entomology and vector control [10].

WHO-GMP also highlighted the key products that they have released since the last MPAC meeting in March 2014. These include: the WHO progress report on the adoption and scaling-up of interventions recommended by WHO in malaria-endemic countries, submitted in response to the UN General Assembly resolution 67/299 on consolidating gains and accelerating efforts to control and eliminate malaria in developing countries, particularly in Africa, by 2015 [11]; a report on the planning meeting for operational research on malaria elimination [12]; a policy brief on the intensified efforts required to withdraw oral artemisinin-based monotherapies from the market, including an overview of WHO’s recommended regulatory actions and country progress to date [13]; a manual for elimination scenario planning which provides a framework to assess scenarios and set realistic timelines for moving towards elimination based on programme coverage and funding [14]; and, a report on the safety and effectiveness of single-dose primaquine as a *P. falciparum* gametocytocide [15].

Also mentioned in brief were the results from Round 5 of the WHO product testing of Rapid Diagnostic Tests (RDTs) [16] and an update on artemisinin resistance [17] – both of which were also agenda items at the MPAC meeting and so have separate sections devoted to them in this meeting report.

In addition, WHO-GMP provided MPAC members with the results of an online survey to seek feedback on the MPAC framework, which drew 123 responses from a variety of stakeholders worldwide [9]. The results indicated some areas for improvement, such as additional avenues for policy dissemination, but overall appreciation for the strengthened policy making process. MPAC and WHO-GMP thanked the global malaria community members who responded to the survey, and also those who provide regular informal feedback and suggestions via the WHO-GMP Secretariat. MPAC members thanked the acting Director, Dr. John Reeder, for his efforts to assure a harmonious transition and looked forward to the incoming leadership of Dr. Pedro Alonso, who assumed the post of Director WHO-GMP in mid-October 2014 [18].

### Update on drug resistance and containment

The chair of the Drug Resistance and Containment (DRC) Technical Expert Group (TEG) updated MPAC on the TEG’s 28-30 April 2014 meeting in Geneva [19]. The information presented included an update on what was known at the time about Kelch 13 mutation which has been associated with delayed parasite clearance. Presence of these mutations is being considered in the development of new definitions of suspected and confirmed artemisinin resistance (see DRC TEG meeting report [20] for more details). The majority of the TEG update was focused on the feasibility of elimination in the Greater Mekong Subregion (GMS) as a strategy to contain multi-drug resistance [21].

The DRC TEG confirmed that *P. falciparum* resistance to artemisinin has emerged independently in multiple geographic areas within the GMS, thus raising concerns about the effectiveness of a “firewall approach”. At the border between Cambodia and Thailand, *P. falciparum* is resistant to almost all available antimalarial drugs. Although great progress has been achieved recently in reducing the *P. falciparum* malaria burden in the GMS through aggressive malaria control measures, this progress is being threatened by the emergence of multi-drug resistance.

Based on the analysis of the DRC TEG, MPAC recommended to WHO-GMP that it adopt the goal of elimination of *P. falciparum* malaria in the GMS by 2030 to counter the threat of multidrug resistance, including artemisinin resistance, and to prevent its spread. Based on a feasibility exercise commissioned by WHO-GMP, *P. falciparum* elimination in the GMS appears to be technically and operationally feasible at a reasonable cost, and is in line with the elimination goals of GMS countries themselves. It should, therefore, be strongly supported and pursued urgently while currently available tools remain effective. MPAC supported the adoption of this goal by affected countries in the GMS.

MPAC recommended that in order to achieve the goal of elimination of *P. falciparum* malaria in the GMS by 2030, an effective joint sub-regional governance structure that clearly delineates roles and responsibilities of GMS countries, WHO, and other partners must be established. An enabling environment must include strong country leadership, political commitment at all levels, and sustainable resource mobilization based on an agreed strategy. Success will also require enhanced involvement of the private sector, and an ongoing coherent research agenda to inform and enhance elimination efforts. In addition, it will be necessary to try out and validate novel interventions, several of which have been identified.

WHO-GMP will liaise with the WHO South East Asia and Western Pacific Regional Offices to support the preparation of an elimination strategy under the coordination of the Emergency Response to Artemisinin Resistance in the GMS (ERAR) hub and the leadership of the GMS countries and in collaboration with partners. WHO-GMP will update MPAC on progress made at its next meeting in March 2015.

### Control of residual malaria parasite transmission

The Malaria Vector Control TEG (VC TEG) presented one of the main outputs from its 24 – 26 February 2014 meeting in Geneva– a review and associated guidance on the control of residual malaria transmission by mosquitoes whose behaviour, such as outside biting, makes them poorly susceptible to control through core malaria vector control interventions [22].

The current core malaria vector control interventions are LLINs [23] and IRS [24], with larval source management applicable in certain settings where mosquito breeding sites are few, fixed and findable [25]. LLINs reduce malaria parasite transmission mainly by killing or blocking mosquitoes that attempt to feed upon humans under nets. IRS kills mosquitoes and reduces longevity when they rest on insecticide-sprayed surfaces inside houses or other structures, usually after feeding on their occupants. However, the effectiveness of both of these interventions relies on a number of factors that include susceptibility of mosquitoes to the insecticides used, adequate coverage rates, quality and timely implementation, and user acceptance and compliance.

While the factors that can limit the effectiveness of existing interventions are important and require due attention, evidence from a variety of settings indicates that residual malaria parasite transmission occurs even in areas with good access to and usage of LLINs or well-implemented IRS [26]. Such transmission is maintained due to a combination of human and vector behaviours, for example when human populations reside in or visit forest areas, or are exposed outside houses during mosquito biting times; or when local mosquito vector species avoid core interventions, for example by resting outdoors away from indoor treated surfaces. In many malaria-endemic areas, it is likely that this residual transmission will preclude malaria elimination in the absence of new vector control interventions.

Technical guidance presented by VC TEG and endorsed by MPAC was for national malaria control programmes to prioritize the implementation of current tools whilst improved or novel vector control interventions are under development and validation. Potential interventions identified were those that:

1. Exclude or deter indoor entry using physical screening barriers or repellents;
2. Following entry, prevent successful indoor feeding and/or resting using exit or other barriers, repellents, or insecticides with no deterrent properties;
3. Prevent successful outdoor feeding by using insecticide-treated clothing or repellents which directly protect people;
4. Reduce adult vector densities or transmission potential by outdoor attractants which lure and trap/kill mosquitoes, topical or systemic insecticides for livestock that kill zoophilic mosquitoes during or after feeding, application of insecticides to natural sugar sources or by introducing insecticidal sugar baits.

Robust entomological surveillance-response approaches are required to characterize the extent and relative contribution of residual transmission to malaria burden across different settings allowing strategy adjustments that possibly include the use of vector control tools beyond the existing core malaria vector control interventions. Once the supporting evidence base for these novel or improved interventions is available, policy setting mechanisms within WHO will make appropriate recommendations for implementation by national programmes.

Based on MPAC guidance, WHO issued the following key recommendations to address residual transmission [27]:

1. National malaria control programmes in collaboration with academic or research institutions should generate local evidence on the magnitude of the problem of residual transmission of malaria, including information on human and vector behaviour, and intervention effectiveness.
2. Industry and their partners are encouraged to develop new vector control tools to address residual transmission. Financial, human and infrastructural resources are urgently needed to support development, evaluation and implementation of such tools.
3. National regulatory authorities should make renewed efforts to ensure that registration processes encourage the rapid availability to the local market of validated new vector control products.

### Progress on the implementation of the global plan for insecticide resistance management in malaria vectors

The Global Plan for Insecticide Resistance Management in malaria vectors (GPIRM) [28] was launched in May 2012 in response to escalating resistance in *Anopheles* mosquitoes. Insecticide resistance has since increased in frequency, intensity and geographical distribution at an alarming rate, especially resistance to pyrethroids in Africa south of the Sahara. At their last meeting in March 2014, MPAC requested WHO-GMP to present an update on the global status of GPIRM implementation.

WHO-GMP reported that some progress has been made in the global implementation of GPIRM [29], including enhanced insecticide resistance monitoring and the establishment of global and regional insecticide resistance databases. Considerable investments in the development of new vector control products have resulted in new IRS formulations, although there still remain only four insecticide classes with two modes of action recommended by WHO for IRS and only one class (pyrethroids) recommended for use in LLINs.

In general, adoption of GPIRM technical recommendations to national policy and operational implementation at the country level has been limited [30]. While some countries have started to use alternatives to pyrethroids for IRS, most have yet to establish and implement national insecticide resistance monitoring or management plans that incorporate sound resistance management practices (such as rotation of insecticides of different modes of action for IRS). This is due largely to major financial, human and infrastructural resource deficiencies and a lack of affordable alternatives to pyrethroids for IRS and LLINs.

MPAC members expressed deep concern over these resource deficiencies and at the worsening extent of insecticide resistance, which threatens the effectiveness of malaria vector control. They requested that WHO-GMP conduct a comprehensive situation analysis and in consultation with malaria endemic countries and their partners prepare a global response plan in order to improve GPIRM implementation, particularly at country level. This should include immediate actions to make available affordable alternatives to pyrethroids for IRS, such as improved global forecasting, pooled procurements, long-term contracts and tax incentives.

MPAC members also indicated that malaria endemic countries need more specific guidance on appropriate vector control, such as clarification of the potential value of the addition of IRS to LLIN for either pre-emptive action against the development of resistance or mitigation of existing resistance, as well as on the recommendation on frequency of insecticide rotation for IRS. In the meantime, countries and their implementing partners should continue to develop and implement national insecticide resistance monitoring and management plans that include alternatives to pyrethroids for IRS. These activities should be incorporated into national malaria control strategies.

### Updates on the global technical strategy for malaria (2016 - 2030), global malaria action plan 2, and the *Plasmodium vivax* technical brief

Following support from WHO Member States at the 2013 World Health Assembly to develop a global malaria strategy for the post-2015 period, and a detailed discussion on an early draft at the March 2014 MPAC meeting, seven regional consultations were held between March and June 2014. The regional consultations gathered input on the initial draft from more than 400 experts representing national malaria programmes, ministries of health, research organizations, and implementing partners. Following the regional consultations, a revised draft was prepared for a web consultation of WHO Member States, consultation participants, and malaria stakeholders that took place in July and August 2014. The process, outlined in the session presentation [31], was led by WHO-GMP and supported by both MPAC and a dedicated Steering Committee of leading malaria experts, scientists, and representatives from malaria-endemic countries.

The result of these many activities is a near-final draft Global Technical Strategy for Malaria 2016-2030 that provides a framework to develop tailored programmes to accelerate progress towards malaria elimination for whole countries and sub-national areas. It defines a clear and ambitious path for endemic countries and global malaria partners, and milestones for the next 15 years up to 2030. It emphasizes the need to achieve universal coverage of the core package of malaria interventions for all populations at risk and highlights the importance of using real-time data for decision-making to drive responses consistent with national or sub-national goals. The draft strategy identifies where innovative solutions will be essential to fully achieve the strategy’s goals, and describes the financial implications of strategy implementation. Importantly, the document also references key WHO-recommended guidance documents and will be updated regularly to incorporate significant innovations in tools and approaches.

MPAC members, having reviewed the near-final WHO technical strategy as part of the background documents for the meeting, expressed appreciation of the inclusive country-driven consultation process that had taken place, and approved the way in which the draft WHO technical strategy is now framed. Further inputs from WHO Regional Offices and the Regional Committee meetings were incorporated before it was submitted for discussion at the 136th meeting of the Executive Board to the World Health Assembly in January 2015. It is expected that the draft WHO technical strategy will be submitted to the World Health Assembly in March 2015, for review as an agenda item at their meeting in May 2015.

Concurrent with the development of the Global Technical Strategy, the Roll Back Malaria (RBM) Partnership has been coordinating the development of the Global Malaria Action Plan 2 (GMAP2) [32]. This action plan, whose draft structure and content were outlined by the co-chair of the GMAP2 Task Force [33], will support the implementation of the draft WHO technical strategy through global advocacy, resource mobilization, partner harmonization, and the engagement of non-health sectors. Both documents are being developed in a collaborative process, involving an overlap in steering committees, and will be jointly launched in 2015 to provide a strengthened platform for continuing malaria investments in the broader post-2015 development agenda. Because GMAP2 is a RBM Partnership document, it will be approved by the RBM Board rather than adopted by the World Health Assembly and its timeframe allows it to complement the final version of the draft WHO technical strategy. A dedicated website for GMAP2 is already collecting ideas [32]; and an on-line general public consultation on the document is expected to begin in early 2015.

WHO-GMP also provided MPAC with an update on the development of the technical brief on *Plasmodium vivax* malaria, which will consolidate all *P. vivax*-specific guidance into one document for the first time [34]. After some discussion on the format and release options for this technical brief, MPAC concluded that the technical brief was a suitable support document for the draft WHO technical strategy, although renaming might be needed to ensure it is not confused as a separate strategy. MPAC members favoured a 2015 release date, including a closely timed publication of a policy-oriented executive summary and research-oriented journal supplement, before the formal launch of the WHO Global Technical Strategy and GMAP2 later in 2015.

MPAC commended the GTS Steering Committee, the GMAP2 Task Force, and WHO-GMP on progress to date with all three documents, and the leadership of WHO-GMP and RBM on the close alignment of the processes for the draft WHO technical strategy and GMAP2. MPAC members expressed sincere thanks to those observers attending the meeting and to malaria stakeholders everywhere for their support of and participation in the consultation process.

### Gaps in current WHO-GMP guidance on malaria elimination

WHO-GMP sought the advice of MPAC on how to address gaps in its guidance to countries on achieving malaria elimination [35]. The current WHO field manual on malaria elimination for low and moderately endemic countries [36] was produced in 2007 and has not been updated since then; it will shortly be reviewed. The purpose of the field manual is to inform national governments from endemic countries, partner and donor agencies and field managers about the issues related to malaria elimination, and to serve as a tool in the implementation, monitoring and evaluation of malaria elimination programmes.

Malaria elimination aims for the sustainable interruption of local malaria transmission despite a continued presence of malaria vector mosquitoes and potential importation of parasites through international travel and migration. In areas with intense transmission and extreme poverty, where overall health and development are still weak, the priority is good malaria control using proven tools, such as appropriate case management using accurate diagnostics and effective drugs with artemisinin-based combination therapy, and vector control with indoor residual spraying and insecticide-treated mosquito nets.

Programme reorientation towards elimination may be considered in areas where essential clinical services are available, the basic needs of the population are covered, malaria transmission has been reduced to a level where less than 5% of all febrile patients suspected of having malaria carry malaria parasites, and case-loads are becoming manageable. WHO-GMP explained that the aim in the pre-elimination phase is to set up the quality-controlled systems required for the elimination phase, which should then be fully implemented when malaria incidence has been reduced to less than 1 infection per 1000 people at risk per year.

At present, WHO-GMP grants certification of malaria elimination to countries that have interrupted local transmission for a period of three or more years and have high-quality surveillance systems that provide credible evidence supporting the absence of ongoing local transmission. However, WHO-GMP asked MPAC and those observers present at the MPAC meeting if the scope of the field manual should be expanded by omitting the specification “for low and moderate endemic countries”.

MPAC members suggested that WHO-GMP should conduct a stakeholder survey similar to that which supported the new edition of the WHO Guidelines for the Prevention and Treatment of Malaria (MTGs). The subsequent changes required to the field manual, if any, will determine the need to convene an Evidence Review Group (ERG) to expand the field manual’s scope.

WHO-GMP also proposed an ERG on Mass Drug Administration (MDA- all the people in a broad geographic area are given malaria drugs without any screening), Mass Screening and Treatment (MSAT – all the people in a broad geographic area are screened, regardless of whether they have symptoms of malaria), and Focused Screening and Treatment (FSAT –screening all the people in a defined geographical area and providing treatment for those who test positive) [37,38]. MDA has received renewed interest over the last decade in the context of malaria elimination initiatives and as part of artemisinin resistance containment efforts. In 2010, WHO held a consultation which reviewed some of the past experience with MDA and screening programmes, their potential advantages and disadvantages, and highlighted the need for more research to define better their potential role in malaria control, in particular in elimination programmes.

Now that new research has accrued, MPAC approved the establishment of an ERG on the role of MDA, MSAT and FSAT for malaria transmission reduction and elimination to:

- review all available published and unpublished reports on the impact of MDA, MSAT and FSAT on malaria transmission, building on a recent Cochrane Review and an additional review from Global Health Group;
- review results of experiences/unpublished studies of large-scale implementation of MDA in Comoros, at the Thai-Myanmar border, and Zanzibar; and of MSAT in Zambia and other locations;
- evaluate the additional role of concomitant administration of low-dose primaquine (0.25 mg base/kg) as a gametocytocide for *P. falciparum* together with the artemisinin-based combination therapy (ACT) when deployed for MDA;
- define the specific conditions under which MDA, MSAT and FSAT should be deployed to reduce malaria transmission in terms of endemicity, drugs and dosages, diagnostics, timing and number of MDA rounds, concomitant implementation of vector control measures and best strategies to ensure community uptake and pharmacovigilance;
- identify research gaps and provide recommendations on data requirements, study methods and ethical considerations for research groups and policy makers interested in further evaluating the role of MDA, MSAT and FSAT in reducing malaria transmission.

Originally, WHO-GMP had proposed that the ERG be convened in December 2014, but noted that additional study data would be available by mid-2015. MPAC agreed that the ERG should wait to include the additional data in the evidence review. The ERG will present its findings to MPAC either in March or September 2015, depending on when the evidence review can take place.

### Surveillance, monitoring and evaluation

The Chair of the Surveillance, Monitoring and Evaluation (SME) TEG updated MPAC on its first two meetings (14-16 May and 26-27 August 2014), both of which had taken place since the last MPAC meeting in March 2014.

Much of the SME TEG’s activities so far have involved reviewing the estimation of global malaria indicators, the draft technical strategy for 2016-2030 in which surveillance plays a major role, and indicators for the Sustainable Development Goals [39]. At the country level, the SME TEG has advised WHO-GMP on chemoprevention indicators, and manuals on programme monitoring, health facility surveys, and Malaria Programme Reviews.

In terms of future work, the SME TEG’s immediate priority is to construct and to develop a framework for malaria SME to enable a baseline to be established for monitoring progress against the goals of the draft WHO technical strategy. The TEG also plans to help WHO-GMP create guidance for countries on improving data quality, data capture in the private sector, and capacity building in surveillance and monitoring and evaluation. The TEG’s plans and priorities for its future meetings were strongly welcomed and encouraged by MPAC, as was the opportunity to work closely with RBM’s Monitoring and Evaluation Reference Group (MERG) and others, to avoid conflicting messages to countries and to donors such as the Global Fund. MPAC also encouraged the SME TEG not to focus too heavily on modelling, but to assist countries to improve the quality of their data so that modelling would not be as necessary.

The SME TEG plans to meet twice a year, and will update MPAC on its outputs and outcomes accordingly.

### WHO guidelines for the prevention and treatment of malaria

The Co-chair of the Chemotherapy TEG updated MPAC on progress with developing the third edition of the WHO Guidelines for the Prevention and Treatment of Malaria (MTGs) [40,41]. The MTGs provides comprehensive, global and evidence-based guidelines for the formulation of policies and national guidelines for the treatment of malaria. The Guidelines were first published in 2006 and the second edition was published in 2010 [42]. The MTGs have been produced under the guidance of the TEG on Malaria Chemotherapy.

A draft plan for revision and update of the second edition was presented and endorsed at the meeting of the MPAC in September 2012; updates of the review process had been presented to the MPAC at the March 2013 and March 2014 meetings [2,3,5].

The third edition of the MTGs, as in previous editions, provide guidelines on malaria treatment, including a new section on intermittent preventive treatment, the latest scientific evidence of *in vitro* anti-malarial susceptibility, safety, and the pharmacokinetic and pharmaco-dynamic properties of the different anti-malarial medicines. The guidelines take into account varying levels of malaria drug resistance and background immunity between areas, as well as the operational and feasibility aspects of malaria chemotherapy in severely resource-constrained settings. Based on the results of a recent online survey, during which respondents expressed satisfaction regarding the format of the MTGs, the overall format of the new MTGs has been retained.

The recommendations of the updated MTGs (third edition) were finalized at the last malaria Chemotherapy TEG in June 2014 and were presented to MPAC for ratification. MPAC members approved the recommendations which included prompt diagnosis and effective treatment, intermittent preventive treatment, and modifications to the dosage of anti-malarials for small children. The MPAC expressed concern over the sometimes confusing categories of evidence strength that accompany the MTG recommendations. However, they concluded that changing an internationally recognized, and now WHO-adopted, method of evidence review (The Grading of Recommendations Assessment, Development and Evaluation, otherwise known as GRADE) was beyond their remit. They urged WHO-GMP to make the GRADE categories/nomenclature explicit to countries when disseminating the MTGs, to avoid any confusion about the strength of the MTG recommendations and the evidence base for them.

It is expected that the MTGs will undergo final clearance by the WHO Guidelines Review Committee and other WHO in-house processes by the end of 2014. The third edition of the MTGs is expected to launch in early 2015.

### Round 5 product testing for rapid diagnostic tests

WHO-GMP provided MPAC members with a background and overview of the product testing process, new results from the latest round of testing, and the WHO procurement criteria for rapid diagnostic tests, and an insight on the market trends and impact of repeated rounds of product testing on manufacturers [43].

The results of the latest round of product testing were well received. MPAC members and procurers (namely the Global Fund and US Presidents Malaria Initiative who were present at the MPAC meeting) acknowledged that while product testing remains a voluntary process, with submission of products at the discretion of the manufacturer, there is continued value in the testing programme due to the positive influence it exerts on market quality.

A full copy of the Round 5 Product Testing Report, including an executive summary, is available on the WHO-GMP website [16,44].

### Intermittent preventive treatment and mortality

Intermittent Preventive Treatment in infancy (IPTi) is a malaria control intervention based on the administration of a full therapeutic course of an anti-malarial to all infants at risk of malaria delivered at the time of administration of routine Expanded Programme on Immunization (EPI) vaccines – usually at 10 weeks, 14 weeks, and about 9 months of age. IPTi with sulphadoxine-pyrimethamine (SP) was recommended by WHO in 2010 for areas with moderate-to-high malaria transmission, where resistance to SP is not high [45].

Concern about the absence of an overall effect of IPTi on mortality was raised in 2012 [46]. Noting that a study at Navrongo, Ghana, had found a (statistically non-significant) clustering of deaths in young infants aged 12-14 weeks, who had received SP within the previous month [47], MPAC subsequently recommended that WHO-GMP support a study to analyze mortality data in eight trials which had evaluated the efficacy of IPTi. The findings from this review were reviewed by MPAC. It was noted that the trials that had been conducted did not have the power to detect a small reduction in mortality and members of the committee were reassured that no increase in mortality or clustering in young infants within 30 days after SP administration had been observed in trials other than the one conducted at Navrongo [Aponte *et al*., personal communication].

MPAC concluded that considering the marked, statistically significant protective efficacy of IPTi against all-cause hospital admissions, it is likely that it also decreases mortality, but it is recognized that such an effect has not been documented directly. IPTi with SP can be a cost-effective strategy for reducing malaria morbidity in infants and MPAC urged those countries where IPTI would potentially be beneficial to consider its implementation.

## Discussion

The wording for recommendations were finalized by MPAC during their closed session following the two days of open sessions; conclusions have been included in the summaries of the meeting sessions above, and links to the full set of meeting documents are provided as references.

Position statements and policy recommendations made by the MPAC have been issued formally and disseminated to WHO Member States by WHO-GMP and the WHO Regional Offices. Conclusions and recommendations from MPAC meetings are published in the *Malaria Journal* as part of this series.

Feedback from the MPAC meeting will also be given to and received from the global malaria community at the RBM Board meeting in December 2014, through the publication of this article, and subsequent correspondence.

On-going engagement with and attendance by interested stakeholders at MPAC meetings continues to be encouraged. In addition to open registration for MPAC meetings, which will continue (via the WHO-GMP website starting in January 2015) and attendance by four standing observers (RBM, the Global Fund, UNICEF, Office of the UN Special Envoy for Financing the Health Millennium Development Goals and for Malaria), the active participation of seven rotating National Malaria Control Programme representatives and all six WHO Regional Malaria Advisors adds to the value of these consultations.

## Conclusion

The meeting feedback received from MPAC members, participants and observers [48] was generally positive. Having met six times to date, the format of MPAC meetings and its feedback loops with other advisory bodies and stakeholders is fairly settled. As noted by the MPAC stakeholder survey [9] and as with any policy-setting process, there are some areas where there is room for improvement; these suggestions will be taken into consideration for future meetings and policy dissemination. WHO-GMP and the MPAC continue to welcome feedback, support, and suggestions for improvement of MPAC meetings from the global malaria community. The next meeting of the MPAC will take place from 4 to 6 March 2015 in Geneva, Switzerland. Further information including the agenda and registration details will be made available in January 2015 on the MPAC page of the WHO-GMP website, although questions are welcome at any time [8].

## Endnote

^a^The complete set of all MPAC September 2014 meeting-related documents including background papers, presentations, and member declarations of interest can be found online at http://www.who.int/malaria/mpac/sep2014/en/.

## Abbreviations

ACT: Artemisinin-based combination therapy
DRC: Drug resistance and containment
EPI: Expanded Programme on Immunization
ERG: Evidence Review Group
GMAP: Global Malaria Action Plan
GMS: Greater Mekong Subregion
GPIRM: Global Plan for Insecticide Resistance Management
GRADE: Grading of Recommendations Assessment, Development and Evaluation
IPTi: Intermittent Preventive Treatment in infancy
IRS: Indoor residual spraying
LLIN: Long-lasting insecticide treated nets
MPAC: Malaria Policy Advisory Committee
MTG: WHO Guidelines for the Prevention and Treatment of Malaria
RBM: Roll Back Malaria
RDT: Rapid diagnostic test
SME: Surveillance, monitoring and evaluation
SP: Sulphadoxine-pyrimethamine
TEG: Technical Expert Group
VC: Vector control
WHO-GMP: World Health Organization Global Malaria Programme
